# Cyclic Voltammetry Study and Solar Light Assisted Photocatalytic Activity of the CeFeO_3_/CeO_2_/Fe_2_O_3_ Composite

**DOI:** 10.1002/bio.70009

**Published:** 2024-11-22

**Authors:** M. Kanimozhi, R. Harikrishnan, M. Mani, S. Kumaresan, A. Rajasekar, N. Saradha Devi, Shankara G. Radhakrishnan, L. Sibali, K. Kaviyarasu

**Affiliations:** ^1^ PG and Research Department of Physics Arignar Anna Govt. Arts College Cheyyar Tamil Nadu India; ^2^ Department of Physics St. Joseph College of Engineering Chennai Tamil Nadu India; ^3^ Department of Physics Kalaignar Karunanidhi Government Arts College Tiruvannamalai Tamil Nadu India; ^4^ Environmental Molecular Microbiology Research Laboratory, Department of Biotechnology Thiruvalluvar University Vellore Tamil Nadu India; ^5^ Department of Chemistry University of Pretoria Pretoria South Africa; ^6^ Department of Environmental Sciences, College of Agriculture and Environmental Sciences University of South Africa Florida South Africa; ^7^ UNESCO ‐ UNISA Africa Chair in Nanosciences/Nanotechnology Laboratories, College of Graduate Studies University of South Africa (UNISA) Pretoria South Africa

**Keywords:** microwave‐assisted, oxygen vacancies, photocatalytic activity, rare earth orthoferrite

## Abstract

The semiconducting nature of CeFeO_3_/CeO_2_/Fe_2_O_3_ nanocomposite has permitted the degradation of the organic toxic dye methylene blue under the irradiation of ultraviolet and visible light portions of solar radiation. Fullprof‐assisted Rietveld refinement analysis, performed using the Match software, has revealed the orthorhombic nature of CeFeO_3_. In addition, in the synthesized material, the cubic phase byproduct CeO_2_ was found due to the highly oxidizing nature of the cerium element. This occurred due to 6 min microwave irradiation because the microwave‐assisted technique offered random distribution of heating during the supply of 800‐W power for 30 s, followed by 12 cycles. Additionally, the presence of Fe_2_O_3_ was also confirmed through Match software‐assisted Rietveld refinement analysis through phase matching. During the synthesis, a certain portion of the synthesized CeFeO_3_ experienced overheating, leading to phase transformation from CeFeO_3_ into Fe_2_O_3_ and CeO_2_. The unit cell compositions of CeFeO_3_, CeO_2_, and Fe_2_O_3_ were found in the sample material with 35.54%, 52.43%, and 12.03%, respectively. The appearance of a fingerprint absorption region in the FTIR spectrum around 577.36 and 535.38 cm^−1^ further confirmed the similarity of these values (577.36 and 535.38 cm^−1^) to those obtained from the calculated values obtained by substituting Rietveld refined bond length parameters. The fourth step process in the thermal analysis curve (TGA) revealed the oxidation process, which led to the destruction of the CeFeO_3_ phase, causing the transformation of CeFeO_3_ into two byproducts (CeO_2_ and Fe_2_O_3_). This oxidation process permitted in an observable weight gain, which is observed in the thermal analysis curve (TGA). A cyclic voltammetry study (the experimentally measured current–voltage characteristic curve) revealed slightly distorted semirectangular CV curves, confirming the pseudo‐capacitive behavior of the synthesized composite.

## Graphical abstract

Based on the distribution of variously shaped particles in the SEM images, cubic (CeO_2_), orthorhombic (CeFeO_3_), and Fe_2_O_3_ phase nanoparticles were detected in the synthesized sample. A slightly distorted semirectangular CV curve was observed, confirming the presence of pseudo‐capacitative behavior. Additionally, cathodic peak potentials shift towards more negative values as scan rate (in mV) increases, whereas anodic peak potentials shift towards more positive values.

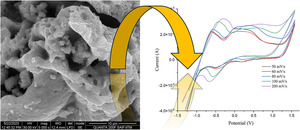

## 1 Introduction

Recently, it has been suggested that the rare earth orthoferrite crystalline phase materials with the general formula RFeO_3_, where “R” is a rare earth element, might be suitable for a variety of applications such as magneto‐optical sensors, tuning of electromagnetic waves, renewable hydrogen energy generation, bioimaging, and photocatalytic activities. This is attributed to the properties of single‐phase orthoferrite crystalline materials with electrical and magnetic features [1, 2]. Due to their high resistivity and low dielectric loss behavior, ferrite is one of the promising materials for use in the telecommunication and electronic industries. They have several industrial and domestic uses across a wide spectrum of frequency [3, 4, 5]. The perovskite rare earth orthoferrite is represented by the common relation RAX3, in which the anion X is filled by oxygen atom and the cations R and A, respectively, stand for the rare earth element and Fe ions [6]. The contribution of magnetic and ferroelectric aspect in RAX_3_ is due to the presence of various A side rare earth element and B side cations. Yuan et al. investigated the spin reorientation temperature and magnetic properties of CeFeO_3_ among rare earth orthoferrites [7, 8]. The unit cell structure of cerium orthoferrite has two different forms of polyhedral arrangements. CeFeO_3_ is forced to exhibit either a weak ferromagnetic property or a paramagnetic behavior due to these two distinct structures, which depends on the magnetic moments of the Ce^3+^ and Fe^3+^ ions, respectively. The unit cell of CeFeO_3_ contains the eightfold coordinated A side and the 12‐fold coordinated B side, which are occupied by cerium and iron ions, respectively. This kind of materials plays a role in many applications such as photo catalytic activity and hydrogen energy generation. Cerium orthoferrite has been demonstrated to exhibit photocatalytic activity in the degradation of Corelene Dark Red 2B and Gentian Violet [9, 10, 11]. According to a previous study, during chemical looping steam methane reforming, it was discovered that the production of hydrogen from CH_4_ occurs when CeO_2_‐Fe_2_O_3_ nanocomposite transforms into CeFeO_3_ perovskite [12]. Several research articles have been published discussing the optical and magnetic characteristics of experimentally synthesized CeFeO_3_. Due to Ce^3+^'s strong oxidation property and its potential to change into Ce^4+^, the production of the CeO_2_ cubic phase is anticipated, making the synthesis of such multiferroic materials challenging [13, 14, 15].

The absence of organic pollutants in the ground water plays a role in maintaining metabolism of living cells. The release of organic pollutants from the textile and paper industries affects the quality of ground water. These pollutants contain complicated aromatic ring structure and possess nonbiodegradable [16, 17]. Therefore, clearing such contamination in ground water is a challenging task. The organic pollutant methylene blue is most used dye in many sectors for the purpose of printing and dyeing. During the process of printing and dying large quantity of methylene blue is discharged to the groundwater. If proper care is not taken, then living animal's health is questionable and may be compromised. Due to the aromatic nature of methylene blue, it is not degradable biologically. Therefore, techniques such as adsorption and chemical precipitation are essentially required to remove organic dye methylene blue in the affected water. However, such methods are believed to be expensive because they may create secondary toxic chemicals. Therefore, the degradation of methylene blue without the creation of secondary toxic chemicals is recommended, and it can be achieved with the help of metal oxides through the oxidation process [18, 19].

In recent studies, the uses and mechanisms of rare earth ferrite materials have been effectively investigated in the degradation of a wide range of organic pollutants such as dyes, pharmaceuticals, and industrial waste. The bandgap of rare earth orthoferrite materials typically falls within the visible light region. An electron–hole pair is created in the unit cell clusters of the rare earth orthoferrite when they interact with visible light from solar radiation. The creation of electron–hole pairs is crucial in initiating the photocatalytic process through redox reactions on the surface of the rare earth orthoferrite unit cell clusters, permitting in the degradation of organic pollutants in wastewater. The presence of the Fe‐O‐RE sublattice facilitates interactions between the A‐site rare earth (RE) cation and the B‐site Fe ion. This interaction plays a significant role in charge separation, thereby improving photocatalytic efficiency. Additionally, it has been shown that the combination of rare earth orthoferrites with metal oxide semiconductors enables enhanced charge separation and utilization of a wide range of the absorption spectrum. In a previous study, rare earth orthoferrite materials such as PrFeO_3_ [20], LaFeO_3_ [21], and CeFeO_3_ [22] were used for degrading organic pollutants. In this study, we use a combination of CeFeO_3_/CeO_2_/Fe_2_O_3_ nanocomposites to degrade methylene blue. The presence of CeO_2_ component plays a crucial role in minimizing the recombination of electron–hole pairs. Its oxygen‐rich active sites stabilize the charges, which helps in prolonging the lifetime of the charge carriers, increasing the efficiency of the photocatalytic process [23].

The synthesis techniques, sintering temperature, chemical composition, cation and anion distributions, and crystallite size affect the properties of rare earth orthoferrite [24]. According to the literature, the production of rare earth perovskite nanomaterials using solid‐state techniques typically demands higher temperatures or prolonged processing times. Temperature up to 300°C is needed for the stable creation of the CeFeO_3_ orthorhombic phase, and above this temperature, the CeFeO_3_ phase may breakdown into byproducts such as CeO_2_ and Fe_2_O_3_ [25]. Therefore, a microwave‐assisted approach is highly suitable for enhancing the creation of the CeFeO_3_ phase, reducing the formation of byproducts. This technique delivers continuous exothermic heating during microwave power supply, enhancing CeFeO_3_ phase production due to optimized reaction kinetics and the creation of various valence states of cations. This enables increased photocatalytic activities for the degradation of organic pollutants. In this experimental study, CeFeO_3_/CeO_2_/Fe_2_O_3_ composite has been synthesized, and their cyclic voltammetry behavior and photocatalytic activity for the degradation of methylene blue have been studied.

## 2 Experimental Procedure

### 2.1 Materials

The necessary chemical compounds (AR grade based) such as cerium (III) nitrate hexahydrate (Ce (NO_3_)_3_.6H_2_O), iron (III) nitrate nonahydrate (Fe (NO_3_)_3_.9H_2_O) and urea distilled water were used for the preparation of the sample material.

### 2.2 Preparation of CeFeO_3_/CeO_2_/Fe_2_O_3_ Composite and Mechanism

CeFeO_3_/CeO_2_/Fe_2_O_3_ has been prepared by adding Ce (NO_3_)_3_.6H_2_O, Fe (NO_3_)_3_.9H_2_O, and urea with the compositions of 1:0.8:2.0, respectively, in the mixed solutions containing 5 mL of deionized water and 0.5 mL of acetone. These solutions underwent the stirring process and then reached the supersaturation stage. The stirred solution was then placed inside a microwave oven and continuously irradiated with microwave electromagnetic radiation for 6 min, during which it was dried through ionic conduction and resistive heating at 800 W of microwave power every 30 s, resulting in the formation of nanoparticles. Due to two phenomena, conduction and resistive heating, the heating process occurring within the sample was observed. Various types of electric polarization are expected to occur continuously within the solution medium when microwave radiation is irradiated on the urea acetone and metal oxide solutions, which contains different kinds of dipolar molecules. During the 6 min of irradiation, the microwave energy is converted into another kind of heat energy. Heating occurs during the polarization mechanism due to the random movement of numerous dipolar molecules colliding with one another. The existence of metal ions in solutions may lead to create electrical current during the microwave irradiation, which results in electrical resistive heating.

## 3 Characterization of CeFeO_3_/CeO_2_/Fe_2_O_3_ Composite

The Bruker D8 advance diffractometer was utilized to acquire the experiment PXRD pattern from Bragg position 20° to 90° and to get numerous features, such as crystalline plane and crystal structure with lattice parameters. An FTIR spectrometer was used to get the fingerprint region of metal oxides, which are main portions of CeFeO_3_/CeO_2_/Fe2O_3_ composite. The fingerprint region contains information about the bond length and mode of vibration of metal oxides. To ensure the crystalline nature and nanoparticle distribution in the synthesized composite, SEM images were used, which was generated by using the SEM instrument (CAREL ZEISS). The measured SEM image contains the outer surface morphology information of the synthesized composite. The photoluminescence spectra of the nanoparticle synthesized under certain experimental conditions will display a distinct band that contains the electronic structure of the sample. PL spectra were obtained using a photoluminescence spectrometer, which sends the beams of light at the CeFeO_3_ nanoparticle sample to excite electrons from the valence band to the conduction band. The lost energy of electrons is released in the form of photoluminescence spectra when these excited electrons return to valance band energy. Thermogravimetry (TGA) measurements were performed with a simultaneous thermal analyzer for characterization of thermal behavior and formation of the CeFeO_3_ nanopowders.

## 4 Results and Discussion

### 4.1 Powder X‐Ray Diffraction and Rietveld Refinement Analysis

**FIGURE 1:**
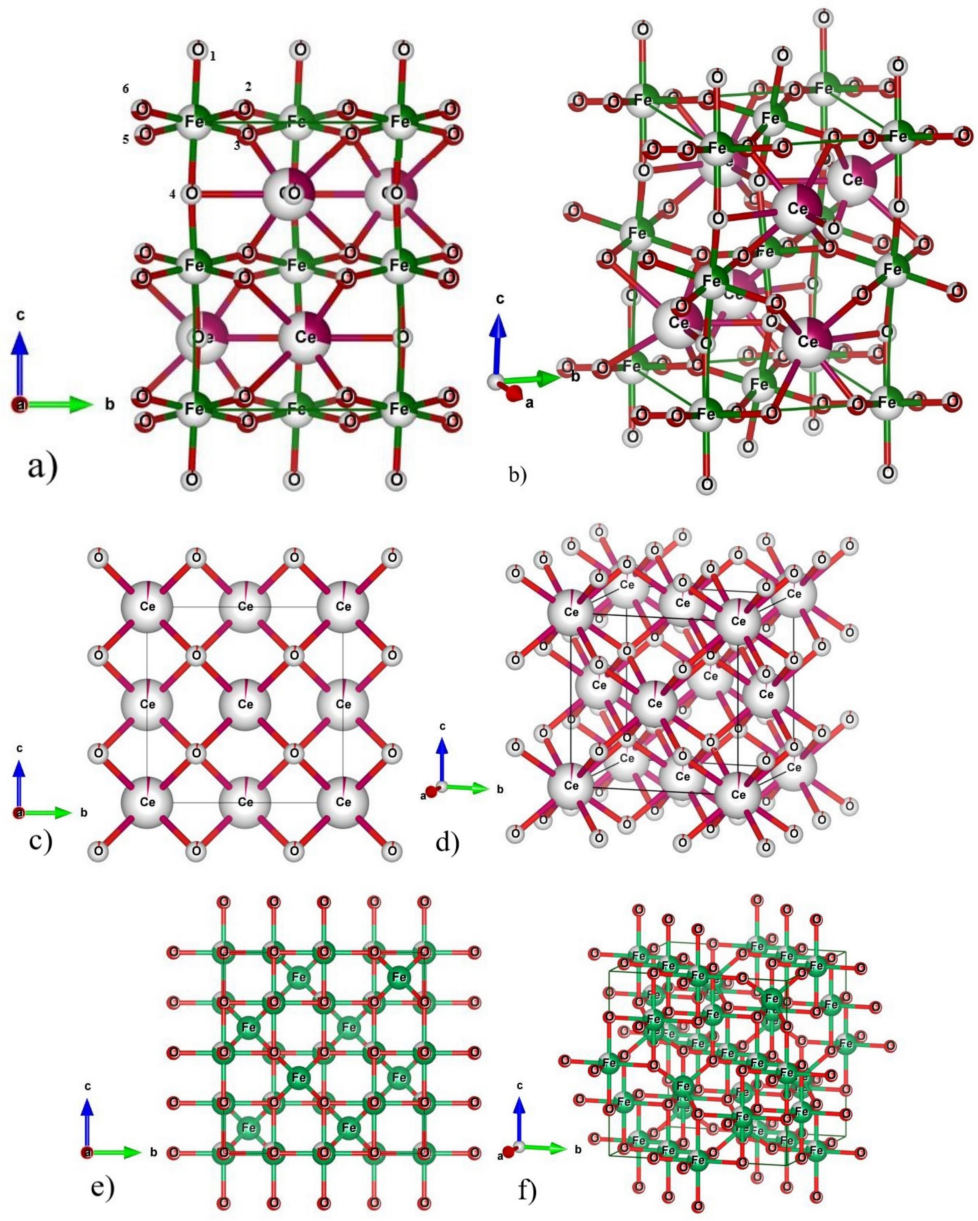
(a) 2D and (b) 3D view of CeFeO_3_, (c) 2D and (d) 3D view of CeO_2_, and (e) 2D and (f) 3D view of Fe_2_O_3_.

**FIGURE 2:**
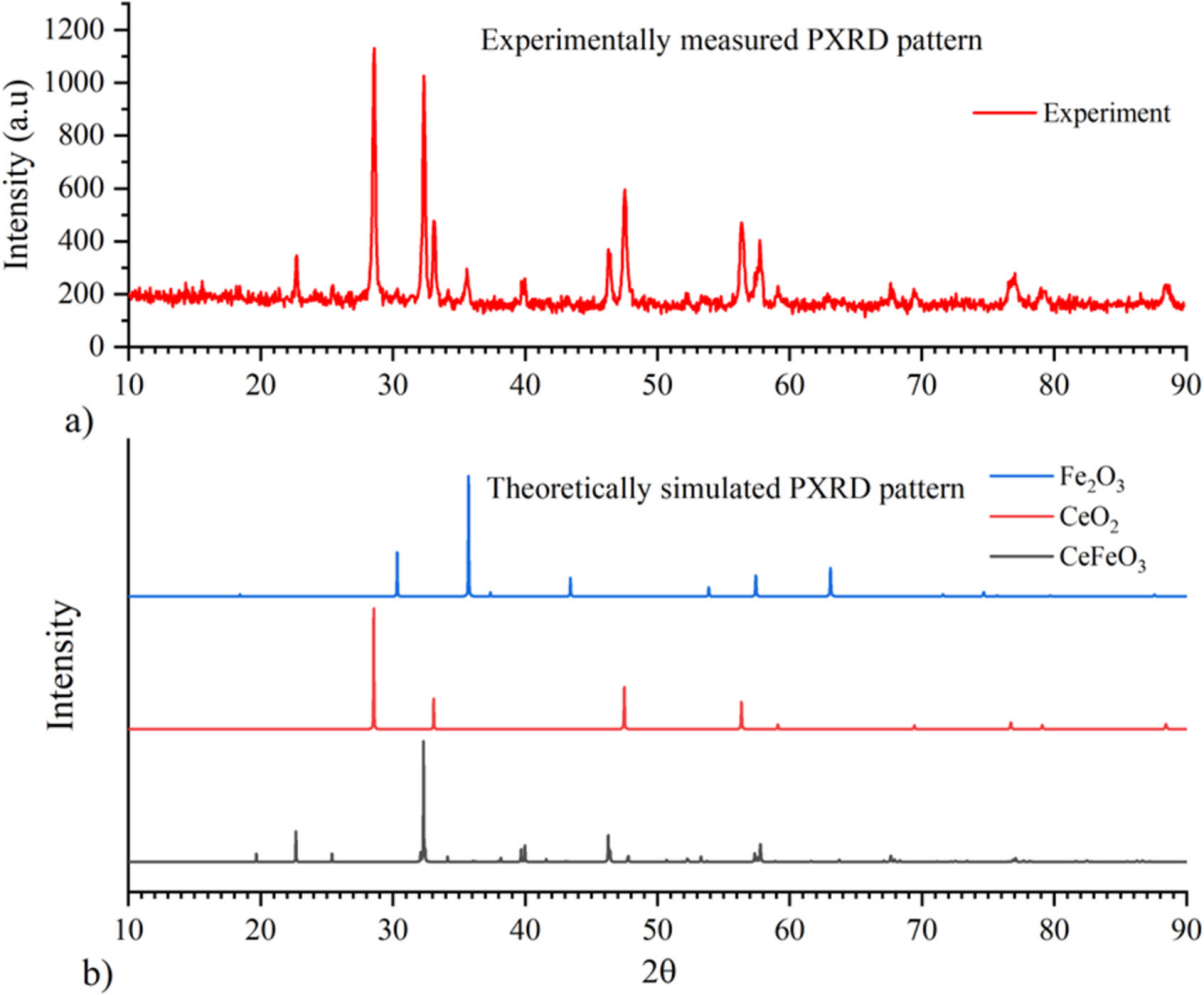
(a, b) The comparison between (a) experimentally observed and (b) simulated (*theoretically*) PXRD pattern of the CeFeO_3_/CeO_2_/Fe_2_O_3_ nanocomposite.

There exists no microscopic device to directly observe the structure of a unit cell with uniformly arranged atoms, but it can be inferred through the Bragg peaks of powder X‐ray diffraction pattern (P‐XRD) with known 2θ values. The measured PXRD pattern indirectly contains information's about lattice or reciprocal lattice planes, the structure of the unit cell, and the location of individual atoms in Cartesian coordinates as shown in Figure 1a–f. The microscopic properties of any synthesized crystalline phase materials can only be determined through the occurrence of diffraction and interference of X‐rays, resulting in a unique PXRD pattern as shown in Figure 2a,b. Unknown parameters, such as lattice constants, vacancies of anions or cations, and space group information of crystalline phase materials, can be calculated by utilizing experimentally measured PXRD patterns, which contains distinct peak positions and intensities. Using computational Fullprof and Match (V3) software packages, microscopic parameters of unit cells of any crystalline phase materials can be easily identified [26, 27]. The Rietveld refinement software package uses least square approximation techniques for refining the measured PXRD pattern until its intensities and Bragg positions are closely fitted with published or theorical PXRD pattern. The lattice parameters of CeFeO_3_ phase are calculated as *a* = 5.51792 Ǻ, *b* = 5.56306 Ǻ, and *c* = 7.81069 Ǻ. For byproducts CeO_2_ and Fe_2_O_3_, the lattice constants (*a* = *b* = *c*) are calculated as 5.40750 and 8.35985 Ǻ, respectively, which are tabulated in Table 1.

**TABLE 1:** Structural parameters of synthesized cubic and orthorhombic phase (CeFeO_3_).

Formula	Crystalline structure	Space group	Lattice constants		
			*a* (Ǻ)	*b* (Ǻ)	*c* (Ǻ)
CeFeO_3_	Orthorhombic (this study)	P n m a	5.51792	5.56306	7.81069
	Orthorhombic [22]	P n m a	5.51592	5.57332	7.81847
CeO_2_	Cubic (this study)	f m −3 m	5.40750	5.40750	5.40750
Fe_2_O_3_	Cubic (this study)	f d −3 m	8.35985	8.35985	8.35985

**FIGURE 3:**
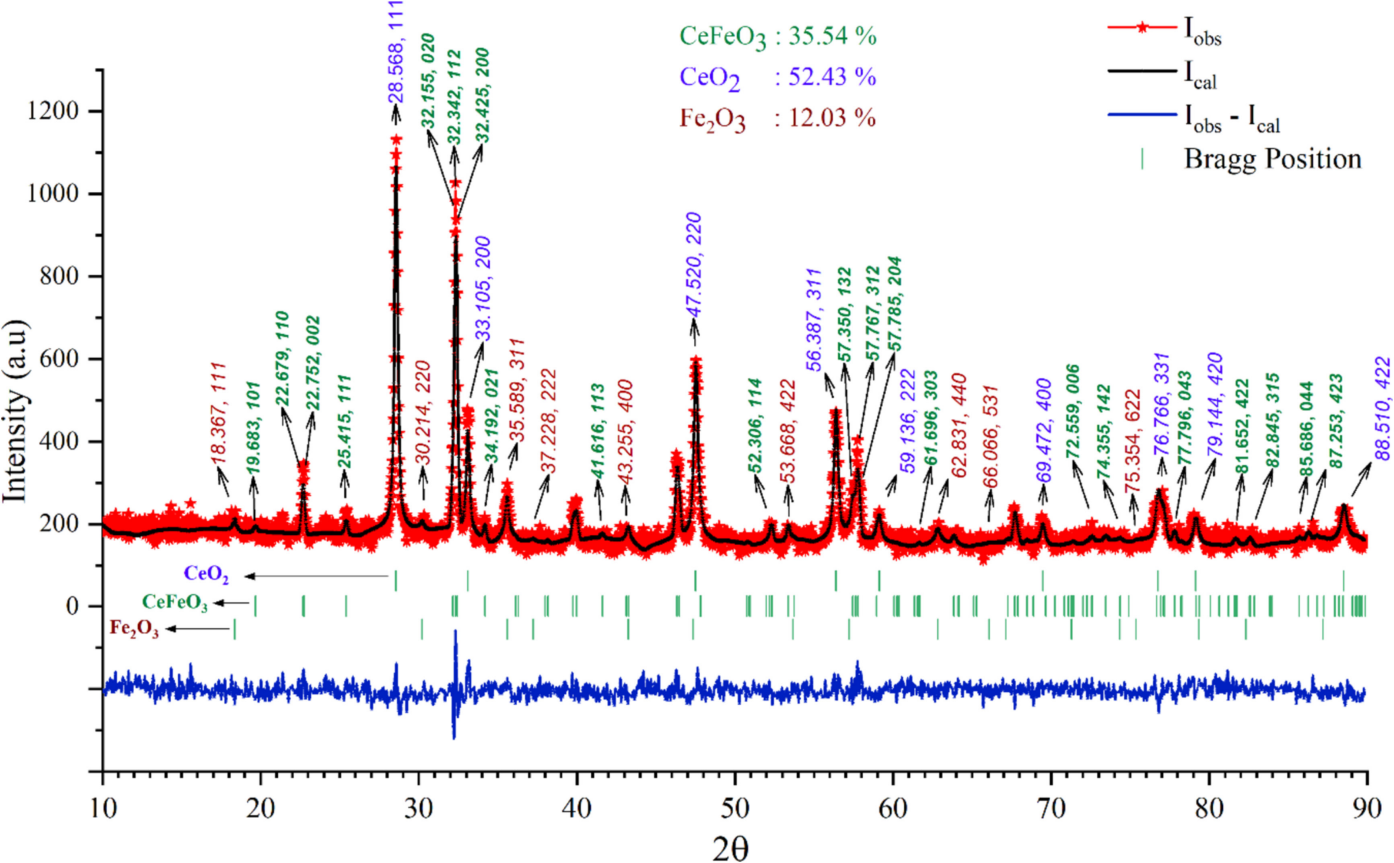
Rietveld refined experimental PXRD pattern of synthesized sample (CeFeO_3_/CeO_2_/Fe_2_O_3_ nanocomposite). The peak positions and Miller indices of CeFeO_3_, CeO_2_, and Fe_2_O_3_ were labeled in green, blue, and red, respectively.

In this study, the Rietveld refinement has been carried out by importing the database entries data_9009008 (CeO_2_) and data_9006316 (Fe_2_O_3_) in the match software. For refining the peaks' position and intensity curves of CeFeO_3_, CIF file format of CeFeO_3_ was used, which was simulated by using the parameters given in Table 2. The CIF file of CeFeO_3_ was created by substituting the known parameters such as atomic coordinate position, crystalline space group information (which is available in Table 2 of previous study titled CeFeO_3_ and ErFeO_3_ composite) [27]. The refined PXRD pattern shown in Figure 3 is obtained by importing all these three CIF files of different crystalline phase materials in the Match software and then performing multiple times of refinement process. From the results of the Rietveld refinement, the presence of CeFeO_3_, CeO_2_, and Fe_2_O_3_ was found in the sample powder with unit cell compositions of 35.54%, 52.43%, and 12.03%, respectively.

**TABLE 2:** Atomic units coordinate locations and occ. factors in the cubic and orthorhombic unit cell box's (*x* = −1, 1; *y* = −1, 1; *z* = 0, 1).

Atom	Coordinate position						
	Previous study			This study			
	*x*	*y*	*z*	*x*	*y*	*z*	Occ.
CeFeO_3_	[27]						
Ce1	0.49390	0.04293	0.25000	0.48975	0.03926	0.25000	0.301
Fe2	0.00000	0.00000	0.00000	0.00000	0.00000	0.00000	0.292
O3	0.58940	0.49192	0.25000	0.58213	0.51535	0.25000	0.045
O4	0.22728	0.28287	0.05096	0.28072	0.24189	0.04331	0.72
CeO_2_	(data_9009008)						
Ce1	0	0	0	0	0	0	0.021
O2	0.25	0.25	0.25	0.25	0.25	0.25	0.042
Fe_2_O_3_	(data_9006316)						
Fe1	0.5	0.5	0.5	0.5	0.5	0.5	0.107
Fe2	0.125	0.125	0.125	0.125	0.125	0.125	0.037
O3	0.25	0.25	0.25	0.25	0.25	0.25	0.236

The higher composition of CeO_2_ in the sample material was achieved due to the highly oxidization nature of Cerium and oxygen environment. In addition, the presence of Fe_2_O_3_ can be expected due to the following reasons. During the synthesis process and microwave irradiation, the burning of metal nitrates and multiple instances of lightning have been observed. The combustion of metal nitrates can generate higher temperatures when irradiated by the microwave region of electromagnetic radiation. It was expected that a certain portion of the synthesized CeFeO_3_ sample experienced overheating, leading to higher temperatures. This increase in temperature, along with the oxygen‐rich environment, resulted in the transformation of CeFeO_3_ into CeO_2_ and Fe_2_O_3_. Consequently, a small portion of Fe_2_O_3_ was found in the composite materials. According to a previous study [28], at higher temperatures, due to the oxidation process, CeFeO_3_ undergoes transformation into by‐products such as CeO_2_ and Fe_2_O_3_. Typically, the oxidation process causes weight gain in the material. The fourth step process (Figure 5) in the thermal studies confirmed the weight gain in the prepared sample, indicating the oxidation process and the transformation of CeFeO_3_ into CeO_2_ and Fe_2_O_3_.

### 4.2 Fourier Transform Infrared Analysis (FTIR)

By absorbing energy from the infrared (IR) frequency region of photon, molecule (reduced mass system) at the atomic scale level undergoes vibrational transitions, when the natural frequency of the atomic molecule matches with the photon frequency. This creates the absorption of IR radiation and the generation of fingerprint spectra for the sample material. These spectra are highly dependent on the chemical composition and band configuration. Hook's law gives the relationship between frequency of vibrating metal oxide ($\bar{\nu }$), reduced mass (μ), and force constant. Hence, it is used to calculate the natural vibrational frequency of harmonically vibrating metal oxide groups [27].

(1) $$\bar{\nu }=\frac{1}{2\mathrm{\pi c}}\sqrt{f_{c}\mu^{-1}}$$

According to published research, by observing the vibrational frequency fingerprint region in the FTIR spectrum, one can calculate bond length ($l_{b}$) information using the following mathematical derivation:

(2) $$f_{c}=17{\left(l_{b}\right)}^{-3}\;\left(Nm^{-1}\right).$$

**FIGURE 4:**
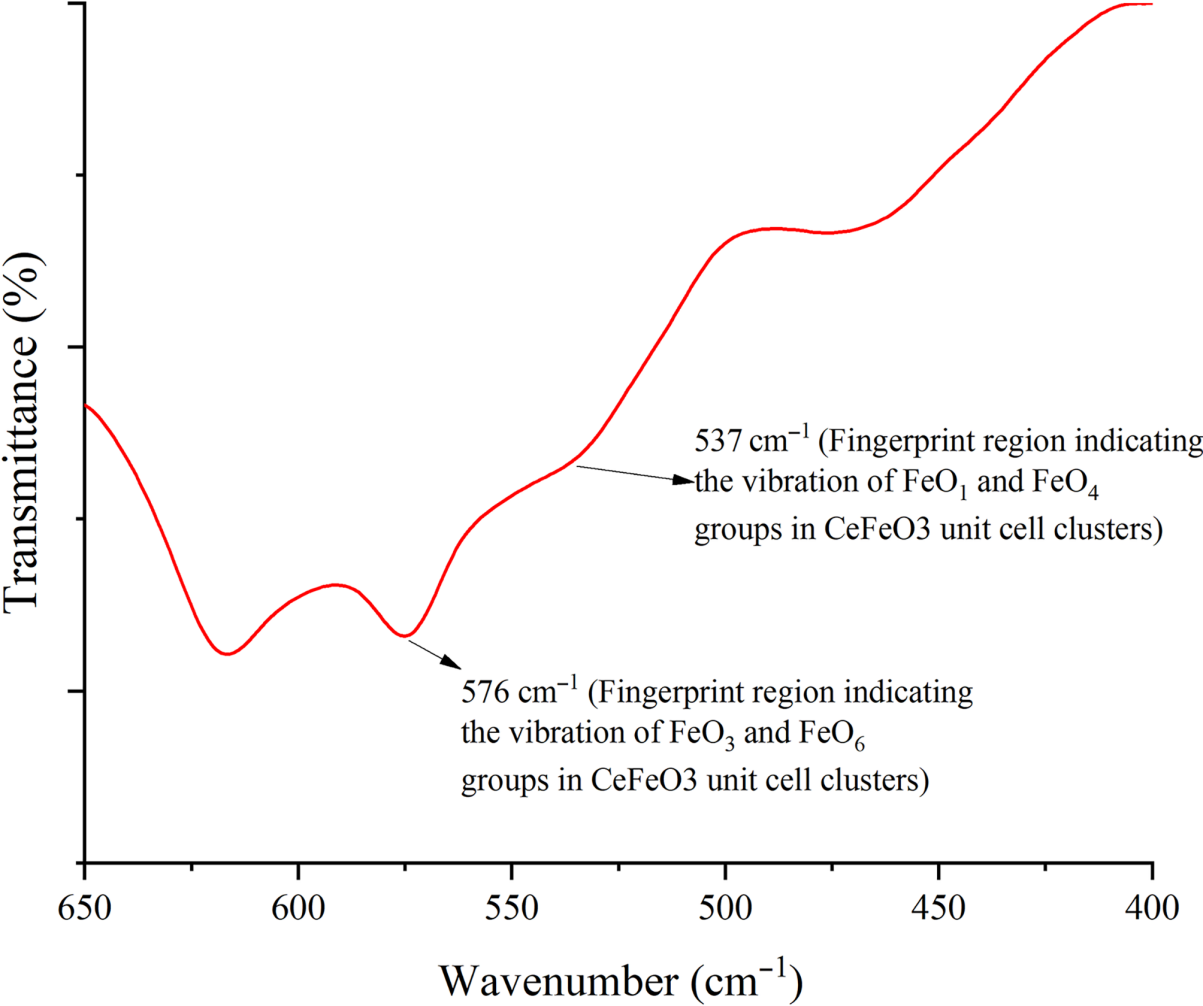
Wavenumber versus transmittance plot (*FTIR fingerprint region*) of Fe‐O groups in the unit cell clusters.

In addition, the natural vibrational frequency of the metal oxides can also be calculated using above equation by inserting the known bond length (*l*_*b*_) value of metal oxide groups as shown in Table 3. In this study, the bond lengths of Ce‐O and Fe‐O are extracted from VESTA software by inputting the calculated Rietveld refined parameters. By using the information given in equation, the vibrational frequencies (*in terms of wavenumber*) of (Fe‐O_3_, Fe‐O_6_), (Fe‐O_1_, Fe‐O_4_), and (Fe‐O_2_, Fe‐O_5_) were calculated as 577.36, 535.38, and 507.38 cm^−1^, as shown in Figure 4. During the vibrations, such groups experienced the restoring force constants with 2.44772, 2.10476, and 1.89032 Ncm^−1^, respectively. The calculated results (frequencies), 577.36 and 535.38 cm^−1^, were found to be similar to the observed frequencies in the FTIR spectrum at 576 and 539 cm^−1^, respectively.

**TABLE 3:** The comparison of restoring force constant and vibrational frequencies between Rietveld refined data and FTIR fingerprint regions.

Metal oxides (Fe‐O)		Calculated Bond length (Å)	Reduced mass (μ) 10^−26^ kg	Calculated Force constant (Ncm^−1^)	Vibrational frequency (cm^−1^)		Force constant (From FTIR) (Ncm^−1^)	Bond length (from FTIR) (Å)
					Calculated	Observed		
FeO_3_	FeO_6_	1.90793	2.065	2.44772	577.36	576	2.4343	1.9114
FeO_1_	FeO_4_	2.00639	2.065	2.10476	535.38	539	2.1316	1.9979
FeO_2_	FeO_5_	2.07956	2.065	1.89032	507.38			

### 4.3 Thermogravimetry Analysis (TGA)

**FIGURE 5:**
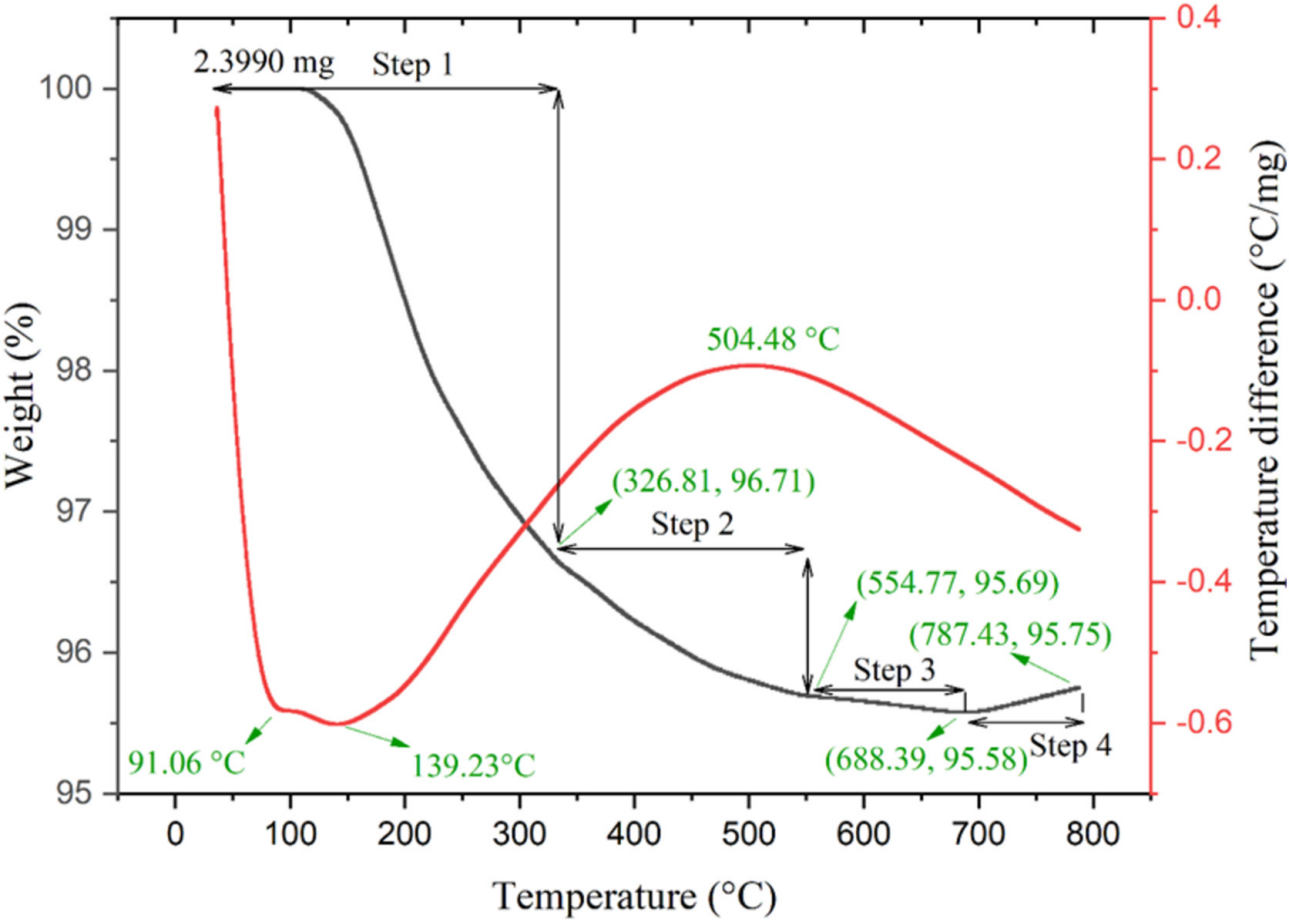
TGA (*black curve*) and DTA (*red curve*) analysis of the synthesized composite.

To identify the temperature range required for stable orthorhombic phase formations and to obtain further details regarding exothermic and endothermic peak temperatures, phase transformation, and oxidation process, thermal analysis was carried out on the synthesized CeFeO_3_/CeO_2_/Fe_2_O_3_ composite using microwave assisted technique. The elimination of impurity functional groups from the sample is much easier at these temperatures. The reference material and the synthesized material both receive same quantities of thermal energy during the Differential Thermal Analysis (DTA) of the composites [29]. In Figure 5, the graph shows experimentally measured TGA (black curve) and DTA curves (red curve) of a synthesized CeFeO_3_/CeO_2_/Fe_2_O_3_ composite. The black curve reveals a four‐step weight loss process that occurs during heating of the sample material from room temperature to 800°C. The initial stage involves weight loss primarily driven by the elimination (evaporation) of lighter elements. As the temperature increases, in the second stage, the evaporation of nitrates takes place. This is a result of the combustion process involving metal nitrates because incompletely burned composition of metal nitrates still exists in the sample material during the microwave power supply. Moving to the third step, the evaporation process targets the firmly bonded groups surrounding the surface of CeFeO_3_ and CeO_2_ unit cell clusters. Finally, the oxidation process takes place in the fourth step process, resulting in an observable increase in weight. A previous study concluded that the CeFeO_3_ phase destruction occurs (*oxidation process*) at higher temperatures and CeFeO_3_ transforms into CeO_2_ and Fe_2_O_3_, as indicated by relation.

(3) $$6\text{CeFe}O_{3}+O_{2}=6\mathrm{Ce}O_{2}+2{\mathrm{Fe}}_{2}O_{3}$$

During the oxidation process, weight gain occurs because of increased oxygen content. Similarly, the weight gain in the TGA curve of CeFeO_3_/Fe_2_O_3_ composite was observed in the previous study [28]. Two endothermic peaks were observed at 91.06°C and 139.23°C, indicating the presence of lighter element. Additionally, the exothermic peak was observed at 504.48°C, suggesting the release of thermal energy which typically occurred due to combustion of metal nitrate compounds. In a previous study, the thermogravimetric analysis results showed an exothermic peak at 490°C for the synthesized CeFeO_3_/ErFeO_3_/CeO_2_ composite [27]. This result is similar to the observed exothermic peak at 504.48°C in this study because the same microwave assisted technique has been carried out in a previous study [27].

### 4.4 Photoluminescence Spectrum Analysis

**FIGURE 6:**
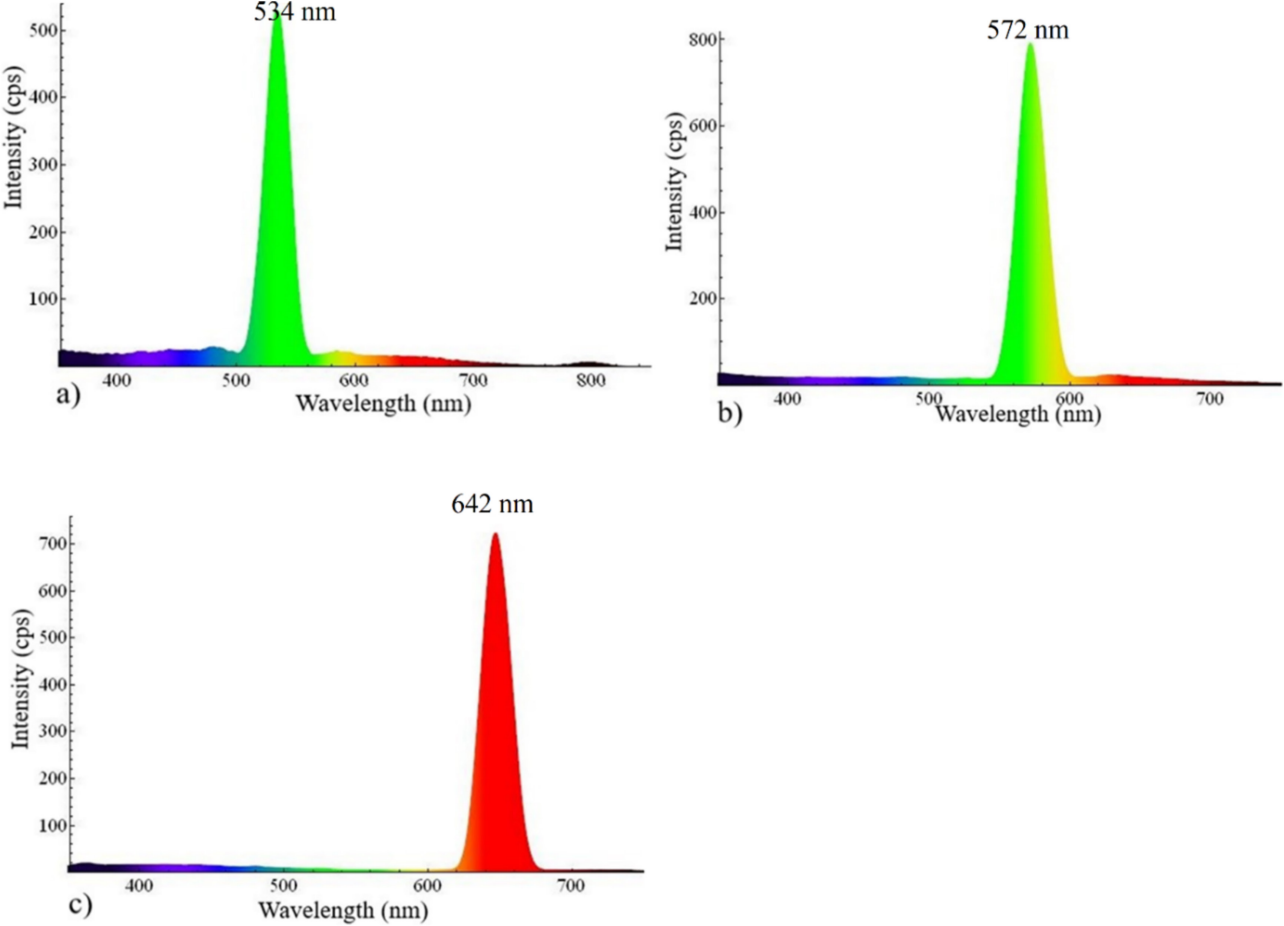
Fluorescence emissions of synthesized CeFeO_3_/CeO_2_/Fe_2_O_3_ composite excited at wavelengths (a) 260, (b) 280, and (c) 320 nm, respectively.

When electromagnetic radiation excites an electron, it undergoes a transition from the valence band to the conduction band and when it returns to the top of the valence bond. The excited electron no longer remains in the lower or high energy state of conduction band. If the excited electrons make the transition from a higher conduction band to a lower conduction band without emitting radiation, this is known as a nonradiative transition, and the lost energy is then transformed into phonons. The excited electron produces the photoluminescence spectrum through the radiative process when it jumps from the conduction band into the valance band through the radiative process since the lost energy is occupied in the form of light (photoluminescence spectrum) [30, 31, 32, 33]. The measured photoluminescence spectrum of the CeFeO_3_/CeO_2_/Fe_2_O_3_ composite is shown in Figure 6. Upon irradiating the material with incident radiation at wavelengths of 260, 280, and 320 nm, the nanocomposite exhibited emission peaks at 534, 572, and 642 nm, respectively. The emission peaks at 534 and 572 nm are associated with green emissions, whereas the peak at 642 nm is assigned to red emissions. Each peak in Figure 6 displays a sharp and uniform curve, confirming the homogeneous distribution of nanoparticles in the CeFeO_3_/CeO_2_/Fe_2_O_3_ composite because SEM images reveal the nanoparticles distributions homogeneously. Broadening occurs in fluorescence emission peaks is typically explained by the fact that in the synthesized composite, nanoparticles are distributed with different sizes. Such particle size affects the bandgap energy of a nanoparticle because the bandgap of the nanoparticle is defined by the principles of quantum confinement theory.

This principle states that the bandgap of nanoparticles increases when their particle size is reduced. As the nanoparticle size decreases, the positively charged holes (*valance band*) and negatively charged electrons (*conduction*) occupy the tiniest region in space and are squeezed nearer to each other. Hence, higher energy of photon is required to trigger the excitation process to overcome Coulomb attractive forces within the quantum confined well. Therefore, the broadening is observed in the emission peaks because the synthesized composite contains CeFeO_3_/CeO_2_/Fe_2_O_3_ nanoparticles with various sizes. One can avoid broadening in the emission peaks by synthesizing nanoparticles with the same size distributions. The emission peaks at 534, 572, and 642 nm are directly related to the bandgaps of 2.32, 2.17, and 1.93 eV, respectively. These emission peaks allow the CeFeO_3_/CeO_2_/Fe_2_O_3_ composite to utilize a broad range of the solar spectrum, including the visible portion, to generate excited electron–hole pairs, thereby improving the efficiency of its photocatalytic behavior in degrading methylene blue. This fluorescence behavior of CeFeO_3_/CeO_2_/Fe_2_O_3_ composite permits the application of degradation of toxic pollutants from the polluted water under sunlight radiations.

### 4.5 SEM and EDAX Analysis

**FIGURE 7:**
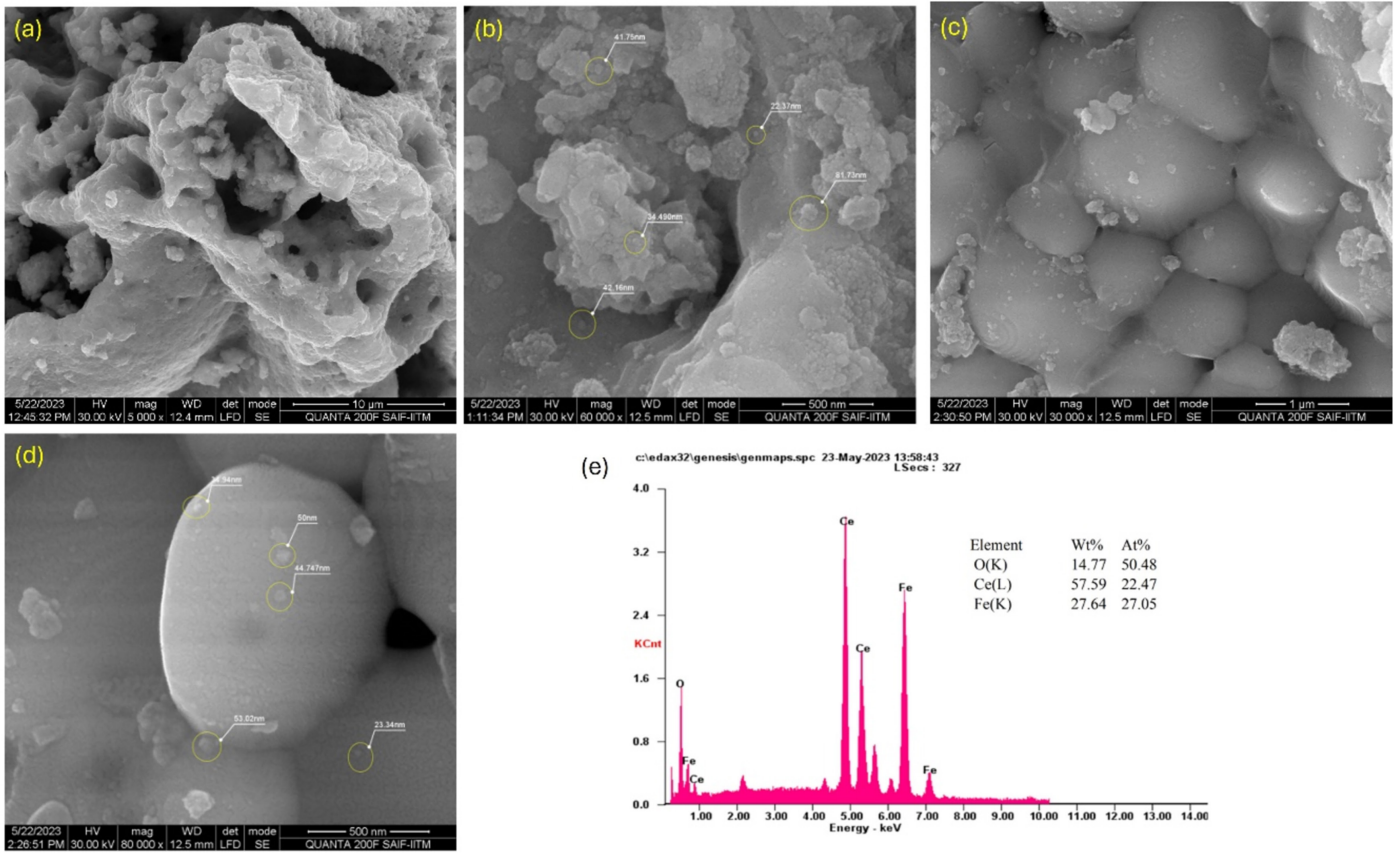
(a–e) SEM and EDAX spectrum of CeFeO_3_/CeO_2_/Fe_2_O_3_ nanocomposites.

The scanned morphologies of CeFeO_3_/CeO_2_/Fe_2_O_3_ nanocomposite, with various magnifications ranging from 200 to 500 nm, are shown in Figure 7a–d. This figure demonstrates the presence of nanoparticles with variously shaped distributions. The presence of cubic (CeO_2_) orthorhombic phase (CeFeO_3_), Fe_2_O_3_ phase structured nanoparticles in the synthesized sample was confirmed by looking at the distribution of variously shaped particle in SEM images. SEM instrument collects the morphologies information of the synthesized composite by making the interactions between the wavelike nature of fast‐moving electrons and the exterior surface of CeFeO_3_/CeO_2_/Fe_2_O_3_ nanoparticles [34]. From the SEM images, typical sizes of nanoparticles were discovered to be in the nanometer range. With magnification ranging from Figure 7a–d, it depicts the exterior surface of CeFeO_3_/CeO_2_/Fe_2_O_3_ composite. These pictures show that there are spherical particles in nanodimensions dispersed throughout the composite material. A yellow circle was drawn around a few small, spherical particles, and it includes a list of the various particles size that were correspondingly determined between 22.37 and 81.73 nm. The weight percentages of the Ce, Fe, and O atoms are displayed in the EDX Spectrum of CeFeO_3_/CeO_2_/Fe_2_O_3_ in Figure 7e. The synthesized nanocomposite material is exposed to fast moving electrons to measure the EDS spectra, because each element's energy level diagram is distinct and each emitted X‐ray wavelength is directly related to the energy level spectrum of Ce, Fe, C, and O elements. The L series element Ce is identified by the peaks at 0.6, 5.05, 5.4, and 6.6 KeV in the EDX spectrum. K series Fe is responsible for the creation of the remaining peaks.

### 4.6 Cyclic Voltammetry Studies

Cyclic voltammetry is one of the electrochemical characterizations of synthesized materials, which measures the current when voltage is given. This technique uses potentiostat for varying the input potentials. It has been reported that the bandgap, particle size distributions, the presence of defects, and the composition of different elements affect the voltametric retaliation of the synthesized semiconducting materials [35, 36, 37, 38].

**FIGURE 8:**
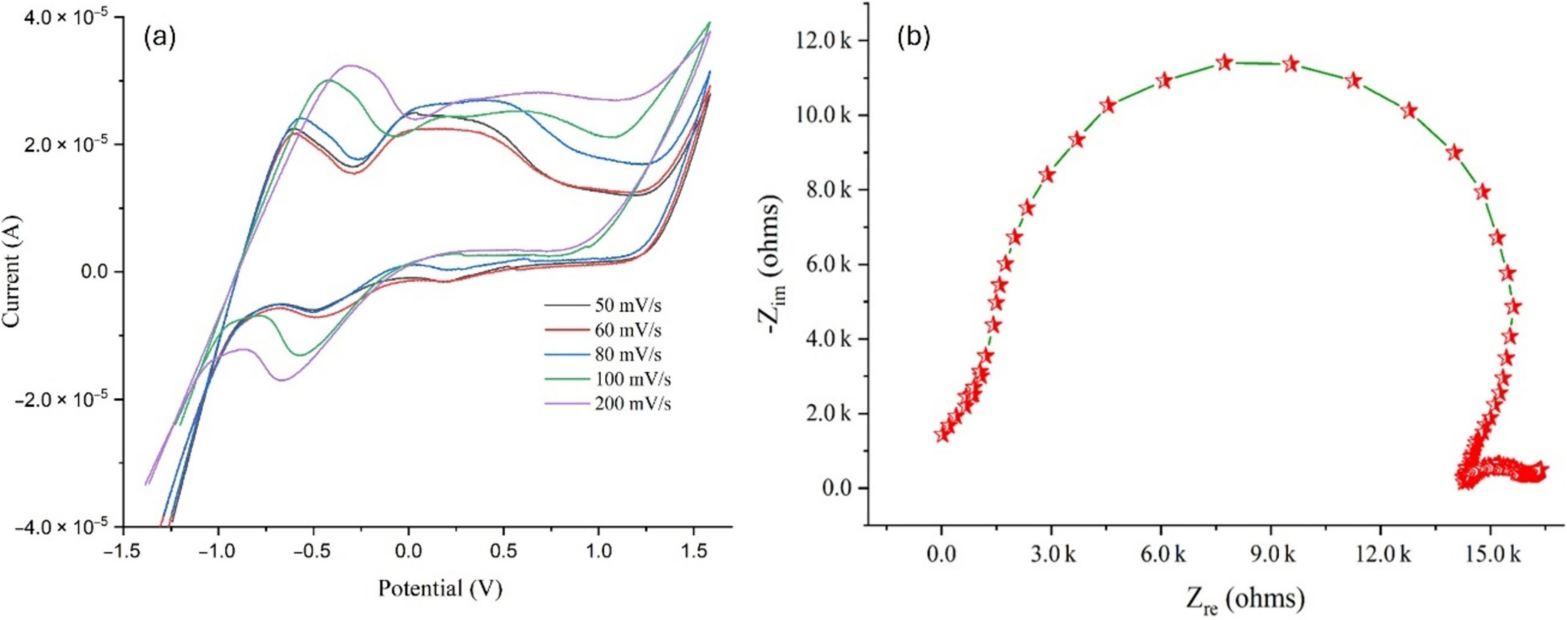
(a). Cyclic Voltammetry studies of synthesized composite; (b) Nyquist plot of CeFeO_3_/CeO_2_/Fe_2_O_3_ composite (*electrochemical impedance analysis*).

In a previous research, cyclic voltammetry studies on the CeFeO_3_ have been studied [39]. Cyclic voltammetry studies of CeO_2_ and Fe_2_O_3_ were reported in a previous works [40, 41, 42]. CeFeO_3_/CeO_2_/Fe_2_O_3_ composite modified glassy carbon electrode was used for electrochemical characterization in this study. The electrochemical workstation contains Tetrabutylammonium percularate (working electrolyte), Glasic carbon (*working electrode*), platinum (*counter*), and Calamol (*reference electrode*). The modified working electrode was immersed, and then, the applied voltage was set and varied between the ranges −1.4 to 1.5 mV with various scan rates (50, 60, 80, 100, and 200 mV/s). In Figure 8a,b, the curves displayed slightly distorted semi rectangular shaped CV curves, confirming the presence of pseudo‐capacitative behavior. As the scan rate (in mV) increases, both anodic and cathodic currents display an increase in magnitude, additionally cathodic peak potentials shift towards more negative values, whereas simultaneously, anodic peak potentials move towards more positive values. This observational result implies that the electrode process is diffusion‐controlled [43, 44]. The modified electrode displayed a pair of redox peaks in the CV curve (scanned at scan rate 200 mV/s) was observed at −0.320 and −0.672 V. These redox peaks typically occur due to oxidation and reduction process. Similarly, the pair of peaks observed in the CV curve scanned at scan rates 100, 80, 60, and 50 V/s are (−0.421 and −0.573 V), (−0.574 and −0.505 V), (−0.601 and −0.502 V), and (−0.612 and −0.498 V), respectively. The impedance spectrum in Figure 8b reveals a well‐resolved semicircle, indicating non‐Debye behavior. Additionally, this plot consists of a noticeable spike at low frequencies. This spike serves as evidence of the electrode polarization effect observed during measurements.

### 4.7 Photocatalytic Study

According to the literature, the typical band gap of CeFeO3 lies between 1.8 and 2.0 eV [45, 46, 47]. This semiconducting nature of the material allows it to absorb visible portions of sunlight, driving the photocatalysis process. The bandgap of CeO₂ is higher than that of CeFeO₃, typically ranging between 2.7 and 3.4 eV [48, 49]. The higher bandgap values of CeO_2_ cause it to absorb ultraviolet portions of sunlight, whereas CeO_2_ with a lower bandgap absorbs visible light radiation, enabling it to initiate the photocatalytic process. Additionally, the bandgap of Fe_2_O_3_ falls within the visible portion of light, allowing Fe_2_O_3_ to act as a photocatalyst in degrading organic dye under sunlight. The combination of these materials (CeFeO_3_/CeO_2_/Fe_2_O_3_) allows for the use of a wide range of solar radiation, enhancing photocatalytic activity through charge separation and transfer. Moreover, CeO_2_ acts as a charge carrier due to its high oxygen storage capacity, which facilitates the stabilization of electron–hole pairs generated in CeFeO_3_ and Fe_2_O_3_. This, in turn, allows for long‐term charge carriers, minimizing the recombination process of electron–hole pairs and improving photocatalytic activity [23, 50, 51, 52, 53, 54].

**FIGURE 9:**
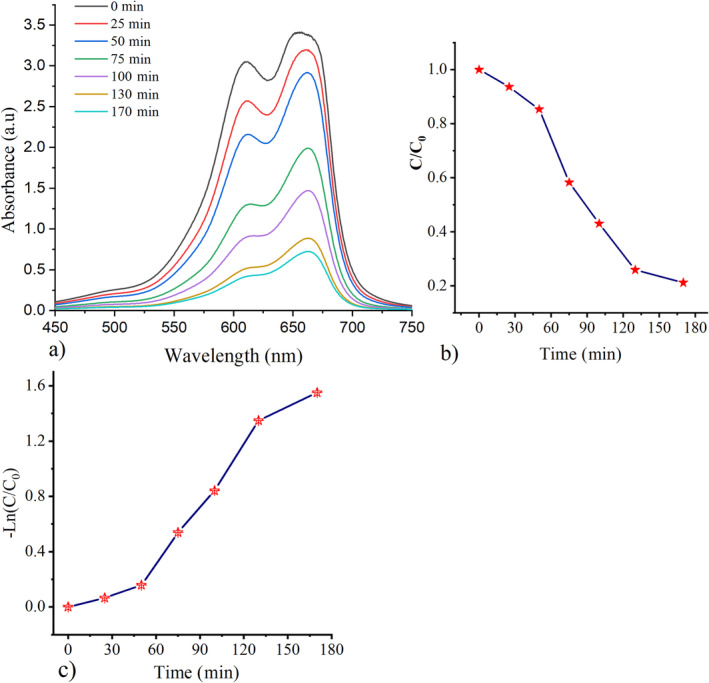
(a) UV‐vis absorbance spectra of stirred solution containing the synthesized CeFeO_3_/CeO_3_/Fe_2_O_3_ nanocomposites and methylene blue, revealing the degradation of methylene blue under sunlight radiations; (b) time versus $\frac{C}{C_{0}}$ plot; and (c) time versus ln ($\frac{C}{C_{0}})$ plot, indicating the rate of degradation of methylene blue.

The catalytic activity of CeFeO_3_/CeO_2_/Fe_2_O_3_ nanocomposite was studied through the mechanism of the photo‐catalysis process under sunlight radiations. For photocatalytic activity, about 6 mg of the synthesized composite was mixed with 100 mL of prepared methylene blue solutions. The methylene blue solution was prepared by mixing 10 mg of methylene blue in 1 L of distilled water, followed by the stirring process. The stirring process was carried out under dark conditions after adding the synthesized composite to the methylene blue solutions. This stirring process permits the nanocomposite to disperse completely in the distilled water. A well‐stirred solution containing CeFeO_3_/CeO_2_/Fe_2_O_3_ composite and MB was placed under sunlight irradiations at 11 Am. Then, stirring process was carried out until the disappearance of blue color of the solutions. The stirred solutions with 0.5 mL were collected after the particular interval period from 11 a.m. to 1.50 p.m. The collected solution was sent to UV–vis absorbance studies to reveal the degradation efficiency. A typical procedure for the degradation of organic pollutants methyl violet (MV), Rhodamine‐B (RhB), and methylene blue (MB) using rare earth perovskites samples was described in the previous studies [55, 56]. Initially, the mixed solutions were stirred in a dark environment and then placed under sunlight radiations. Degradation of the dye occurs under irradiation of UV or visible lights because the sunlight contains UV or visible light radiations. Decolorization of the mixed solution is to be achieved after an interval period. The UV‐vis spectrometer was used to measure the absorption spectrum of solutions. The performance of the degradation of organic dye can be studied using the wavelength versus absorption graphical plot analysis, which is shown in Figure 9.

The degradation of the typical organic contaminant methylene blue under sunlight illumination was known as an effective technique to evaluate the photocatalytic activity of the synthesized CeFeO_3_/CeO_2_/Fe_2_O_3_ composite. As the photocatalytic activity increases, the contaminant methylene blue concentration decreases because the terms *methylene blue concentration* and *photocatalytic activity* are inversely related. Among many metal oxides, the rare earth‐containing perovskite oxide plays a role in the degradation of organic pollutants. The principle involved in the degradation of organic pollutants using rare earth‐containing perovskite (Ce‐FeO_3_) is explained by the following aspects. When sunlight radiation is interacting with the Ce‐FeO_3_ perovskites, electron–hole pair formation occurs, whereas the electron and holes occupy conduction and valance band energies, respectively. Such induced electron lying on the conduction band has the tendency to transfer from Ce‐FeO_3_ unit cell clusters into the surface region of the photocatalyst and could possibly be captured.

For degrading the organic pollutant methylene blue (dye), the following reaction is adopted.

Step I

(4) $$\begin{array}{c} \mathrm{Ce}-{\mathrm{FeO}}_{3}\overset{\mathrm{h\nu}\;\left(\text{photon}\right)}{\to }\;\mathrm{Ce}-\mathrm{Fe}O_{3}\;\left(h^{+}+e^{-}\right), \end{array}$$

where $h^{+}$ and $e^{-}$ denote the photogenerated hole (positively charged) and excited electron (negatively charged), respectively. Photocatalysis process is initiated after the creation of hole pair generations in the mixed solution, where $h^{+}$ and $e^{-}$ act as the oxidizing and reducing agents, respectively.

Step II

(5) dye10toxic agenthvphoton→dye11excitedstate,

(6) dye10toxic agentISC→dye30tripletstate.

Step III

(7) $$h^{+}+{\mathrm{OH}}^{-}\to {\mathrm{OH}}^{*},$$

(8) $$e^{-}+O_{2}\to {O_{2}}^{-*},$$

(9) $${O_{2}}^{-*}+H^{+}\to H{O_{2}}^{*},$$

where ${\mathrm{OH}}^{*}$, ${O_{2}}^{-*}$, and $H{O_{2}}^{*}$ are called as hydroxyl radicals, superoxide radical, and peroxide radicals, respectively.

Step IV

(10)

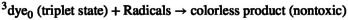




There are four step processes in a typical photocatalysis process in the degradation of organic pollutants. In first step process, electron hole pair generation takes place, which typically occurs in the rare earth containing perovskites under photon irradiation. In second step process, initially, the formation of excited single state of dye occurs and then finally singlet state dye undergoes to triplet state. The third step process involves in the formation of radicals. In fourth step process, the triplet state dye interacts with radicals form colorless product, which is nontoxic. The first, second, and third step process may occur simultaneously, and one cannot achieve degradation of organic pollutants without the first three steps process [57, 58]. In previous studies, CeFeO_3_ [55, 59, 60], CeO_2_ [61, 62], and Fe_2_O_3_ [63] are used as the active catalysts for the degradation of organic pollutants. The types of organic pollutants degraded using CeFeO_3_, CeO_2_, and Fe_2_O_3_ are given in table 4.

**TABLE 4:** Previous research indicating the photocatalytic activity of CeFeO_3_, CeO_2_, and Fe_2_O_3_ nanoparticles for degrading the different organic dyes.

Materials	Source	Organic dye or organic pollutant	Reference
CeFeO_3_	Visible light	Congo red dye	[55]
CeFeO_3_/Graphene carbon nitrate	Solar radiation	Sulfamethoxazole (SMX), atrazine, and bisphenol A	[60]
CeFeO_3_/GO	Visible light	Methylene blue	[47]
CeFeO_3_	Visible light	Methylene blue	[45]
CeFeO_3_	Visible light	Malachite green	[64]
Ni doped CeFeO_3_	Visible light	Methyl orange	[65]
CeFeO_3_/LaFeO_3_/ZnIn_2_S_4_	Visible light	Atrazine	[22]
CeFeO_3_	Visible light	Methylene blue	[59]
CeFeO_3_	Visible light	Coralene dark red 2B	[10]
Co doped CeO_2_	Visible light	Methylene blue, 4‐nitrophenol reduction	[66]
CeO_2_	Sun light	Methylene blue	[67]
Fe_2_O_3_	Sun light	Methylene blue	[68]
Cd doped TiO_2_	Sun light	Methylene blue	[69]
ZnO	Sun light	Methylene blue, Rhodamine B	[70]
ZnO/SnO_2_‐CeO_2_/CuO	Sun light	Methylene blue and methyl orange	[71]

In this study, the synthesized composite material contains CeFeO_3_, CeO_2_, and Fe_2_O_3_ with compositions 35.54%, 52.43%, and 12.03%, respectively. All of them have significantly contributed to the degradation of methylene blue (MB); however, the synthesized material contains CeFeO_3_ and CeO_2_ with the highest compositions. Therefore, the domination of methylene blue degradation by CeFeO_3_ and CeO_2_ is highly expected. The mechanism involved in the degradation of MB by using CeFeO_3_ material is already given in the above paragraphs. The produced positive charge existing in the valance band can interact with the OH‐ or H_2_O species of catalyst surface and can create hydroxyl radicals. In addition, the sunlight‐induced photoelectron present in the conduction band of Fe_2_O_3_ could be transferred to surface molecular oxygen species, as a result the creation of the superoxide radical anion and hydrogen peroxide.

The following equation is used to relate the activity of decoloring efficiency D (%) done by the catalyst (CeFeO_3_/CeO_2_/Fe_2_O_3_ composite) at time *t* [72].

(11) $$D\;\left(\%\right)=\left(\frac{\mathrm{Co}-\mathrm{Ct}\;}{\mathrm{Co}}\right)\times 100$$

Figure 9 displays the measured UV‐vis absorbance spectra of solution containing methylene blue and CeFeO_3_/CeO_2_/Fe_2_O_3_ composite. By using Equation (11), the efficiency of sunlight‐driven photocatalytic activity is calculated as 78.79%, and it took 2 h and 50 min under sunlight irradiation and continues stirring process.

## 5 Conclusion

By using the synthesized CeFeO_3_/CeO_2_/Fe_2_O_3_ composite, the methylene blue degradation efficiency was achieved up to 78.79% under 2 h and 50 min of sunlight irradiation. SEM images confirmed the nanoscale level distribution of CeFeO_3_, CeO_2_, and Fe_2_O_3_ particles. The presence of fingerprint absorption region around 577.36 and 535.38 cm^−1^ confirmed to the similar result of values (577.36 and 535.38 cm^−1^) from the Rietveld refinement analysis. The synthesized composite displayed green and red emission upon striking the photon radiations with wavelengths (260 and 280 nm) and 320 nm, respectively. This emission spectrum reveals the use of wide range of solar spectrum by the photocatalyst CeFeO_3_/CeO_2_/Fe_2_O_3_ in degrading the methylene blue. Weigh gain observed in the TGA curve at above 688°C is due to increased oxygen content when CeFeO_3_ split into CeO_2_ and Fe_2_O_3_ during oxidation process. It was expected that the presence of the CeO_2_ and Fe_2_O_3_ composite in the synthesized material created additional charge separation and transfer, which was believed to increase the efficiency of photocatalytic activity. Moreover, oxygen‐rich active sites around the surface of CeO_2_ facilitated the stabilization of electron–hole pairs typically generated in CeFeO_3_ and Fe_2_O_3_. Cyclic voltammetry study has revealed slightly distorted semirectangular CV curves, confirming the pseudo‐capacitive behavior of the synthesized composite.

## Author Contributions

**M. Kanimozhi:** investigation, writing – original draft, methodology, validation, software, formal analysis, data curation, resources. **R. Harikrishnan:** investigation, validation, visualization, conceptualization, software, formal analysis, data curation, resources, writing – review and editing, methodology. **M. Mani:** conceptualization, investigation, writing – original draft, writing – review and editing, formal analysis, software, data curation, resources, methodology, validation. **S. Kumaresan:** investigation, conceptualization, writing – review and editing, project administration, formal analysis, supervision, resources, software, validation, methodology. **A. Rajasekar:** writing – review and editing, visualization, validation, investigation, data curation, formal analysis, software, methodology. **N. Saradha Devi:** software, formal analysis, data curation, resources, validation, methodology, conceptualization, investigation. **Shankara G. Radhakrishnan:** validation, visualization, writing – review and editing, formal analysis. **L. Sibali:** funding acquisition, validation, visualization, writing – review and editing, formal analysis. **K. Kaviyarasu:** writing – review and editing, visualization, validation, funding acquisition, supervision, resources, project administration, formal analysis, conceptualization.

## Conflicts of Interest

The authors declare no conflicts of interest.

## Data Availability

The datasets generated during and/or analyzed during the current study are available from the corresponding author on reasonable request. All data generated or analyzed during this study are included in this published article.
